# Supramolecular Responsive Chitosan Microcarriers for Cell Detachment Triggered by Adamantane

**DOI:** 10.3390/polym15194024

**Published:** 2023-10-08

**Authors:** Lixia Huang, Yifei Jiang, Xinying Chen, Wenqi Zhang, Qiuchen Luo, Siyan Chen, Shuhan Wang, Fangqing Weng, Lin Xiao

**Affiliations:** 1 Hubei Key Laboratory of Purification and Application of Plant Anti-Cancer Active Ingredients, School of Chemistry and Life Sciences, Hubei University of Education, Wuhan 430205, China; huanglixia@hue.edu.cn (L.H.); jiangyifei2423@163.com (Y.J.); chensiyan0911@163.com (S.C.); wengfq.hue@foxmail.com (F.W.); 2 School of Biomedical Engineering, Shenzhen Campus of Sun Yat-sen University, Shenzhen 518107, China; zhangwq87@mail2.sysu.edu.cn (W.Z.); luoqch3@mail2.sysu.edu.cn (Q.L.); 3 Shenzhen Institute for Drug Control, Shenzhen Testing Center of Medical Devices, Shenzhen 518057, China; wangshuhan@szidc.org.cn

**Keywords:** chitosan, microcarrier, cell culture, cell detachment, host–guest interaction

## Abstract

Supramolecular responsive microcarriers based on chitosan microspheres were prepared and applied for nonenzymatic cell detachment. Briefly, chitosan microspheres (CSMs) were first prepared by an emulsion crosslinking approach, the surface of which was then modified with β-cyclodextrin (β-CD) by chemical grafting. Subsequently, gelatin was attached onto the surface of the CSMs via the host–guest interaction between β-CD groups and aromatic residues in gelatin. The resultant microspheres were denoted CSM-g-CD-Gel. Due to their superior biocompatibility and gelatin niches, CSM-g-CD-Gel microspheres can be used as effective microcarriers for cell attachment and expansion. L-02, a human fetal hepatocyte line, was used to evaluate cell attachment and expansion with these microcarriers. After incubation for 48 h, the cells attached and expanded to cover the entire surface of microcarriers. Moreover, with the addition of adamantane (AD), cells can be detached from the microcarriers together with gelatin because of the competitive binding between β-CD and AD. Overall, these supramolecular responsive microcarriers could effectively support cell expansion and achieve nonenzymatic cell detachment and may be potentially reusable with a new cycle of gelatin attachment and detachment.

## 1. Introduction

Microcarriers are 100 to 300 micron supporting matrices that permit the growth of adherent cells in bioreactor systems. They have a larger surface area to volume ratio in comparison to single cell monolayers, enabling cost-effective cell production and expansion [1,2,3,4]. Microcarriers are composed of a solid matrix that must be separated from expanded cells during downstream processing stages.

Most of the current cellular microcarriers need to harvest cells by tedious downstream operations after cell expansion, and cells adherent to the surface of microcarriers are usually isolated by pancreatic enzyme digestion because microcarriers are mainly used for the culture of anchorage-dependent cells. However, the efficiency of pancreatic enzyme digestion is limited. It is difficult to dissociate cells completely from microcarriers with regular pancreatic enzyme digestion, while overdigestion may cause irreversible damage to cells and reduce total cellular yield and function [5]. Therefore, constructing smart microcarriers with stimuli-responsiveness to achieve nonenzymatic automatic desorption of cells has become one of the main directions of current microcarrier technology. A variety of nonenzymatic responsive microcarriers have been reported during past decades [6,7,8]. As an example, Akbari et al. reported multifunctional temperature-responsive microcarriers (cytoGel) made of an interpenetrating hydrogel network composed of poly(N-isopropylacrylamide) (PNIPAM), poly(ethylene glycol) diacrylate (PEGDA), and gelatin methacryloyl (GelMA) [7]. Cell detachment was achieved by cooling the system to room temperature with up to 70% detachment efficiency. Quantitative analysis with a flow cytometer indicated that more than 90% of cell viability was obtained for these thermally detached cells. Zhao et al. developed near-infrared (NIR) light-responsive graphene oxide hydrogel microcarriers for controlled cell culture and release [8]. After exposure to NIR light, the cell-laden microcarriers underwent rapid shrinkage and the cells were released. The cell release was enhanced as the irradiation power intensity increased. However, to obtain a comparable cell viability with enzyme-digested cells, the power intensity should be lower than 0.5 W cm^−2^ and the irradiation period should be less than 15 s.

Chitosan (CS) is a deacetylation product of chitin. It has good biocompatibility and biodegradability and a variety of biological activities, such as coagulation, antibacterial activity, antitumor activity, and immunomodulatory function. It has the potential to be modified due to the large amount of free amino groups. Based on these advantages, chitosan has been widely used in biomedical fields such as tissue engineering and drug release [9,10,11]. We previously developed highly porous chitosan microspheres as microcarriers for 3D cell culture, in which adhesion and growth of L-02 cells not only took place on their external surface but also within the internal pores of the microcarriers, allowing multidirectional cell–cell interactions [1]. In this work, responsive microcarriers with the abilities of in vitro cell expansion and nonenzymatic detachment were explored based on chitosan microspheres and surface modification. First, chitosan microspheres (CSMs) were prepared through a conventional emulsification crosslinking approach, where glutaraldehyde was used as a crosslinking agent. By crosslinking the amino groups, glutaraldehyde forms covalent bonds that make it difficult for water molecules to penetrate the chitosan microspheres, resulting in increased hydrophobicity and improved mechanical properties. This makes the chitosan microspheres more resistant to harsh environments, such as high pH or temperature, making them ideal candidates for use in various applications, such as drug delivery systems, biomaterials, and tissue engineering [12,13,14]. Second, CSMs were surface-modified with β-cyclodextrin (β-CD) with chemical grafting and subsequently modified with gelatin, which binds β-CD groups via host–guest interactions with its aromatic residues. The modification of gelatin is favorable for cell attachment and expansion when the microspheres are used as microcarriers. Finally, due to the relatively higher affinity of adamantane (AD) to β-CD than gelatin, it is possible to detach the gelatin molecules from the microsphere surface with the addition of AD, which may thus cause detachment of the cells anchored to gelatin. Compared with the reported nonenzymatic responsive microcarriers, this biocompatible microcarrier system is based on gentle supramolecular interactions, enabling cell detachment in a simple and mild manner without changing any external environmental conditions, which is favorable for maintaining cell viability [15,16].

## 2. Materials and Methods

### 2.1. Material

Chitosan (degree of deacetylation > 95%) was purchased from Sigma Aldrich, and its molecular weight (MV) was 1.02 × 10^6^ Da. Surfactants siban 80 (S80), Tween 60 (T60), acetic acid, petroleum ether, glutaraldehyde, and ethanol were purchased from Shanghai Sinopharm Chemical Reagent Co., Ltd.(Shanghai, China), and 1-(3-dimethylaminopropyl)-3-ethylcarbodiimide salt (EDC), N-hydroxysuccinimide (NHS), carboxymethyl β-cyclodextrin (CMCD), gelatin, and adamantane (AD) were purchased from Shanghai Aladdin Biochemical Technology Co., Ltd. (Shanghai, China). All reagents were of analytical grade and used without further purification.

Human fetal hepatocytes L-02 were obtained from Tongji Medical College, Huazhong University of Science and Technology, and cultured in DMEM supplemented with 10% fetal bovine serum and 1% antibiotics (100 mg/mL streptomycin and 100 U/mL penicillin) at 37 °C in a humidified atmosphere containing 5% CO_2_. The cell culture grade reagents used were all procured from Gibico products from Thermo Fisher Scientific (Waltham, MA, USA).

### 2.2. Preparation of Chitosan Microspheres

Chitosan microspheres were prepared with a conventional emulsification crosslinking method [1]. Briefly, 0.6 g of chitosan powder was dispersed in 30 mL of deionized water under magnetic stirring at 400 rpm to form a white turbid liquid. Then, 0.3 g of acetic acid was added dropwise to the suspension to facilitate the dissolution of chitosan, and a transparent 2% (*w*/*v*) chitosan solution was obtained. The chitosan solution was then mixed with 90 mL of petroleum ether containing 4.8 g of Span 80 and 0.2 g of Tween 60. The mixture was subjected to emulsification under stirring at 400 rpm at 40 °C for 2 h, after which 1.5 mL of 25 wt% glutaraldehyde aqueous solution was added to the emulsion and crosslinked for 3 h. The crosslinked emulsion was then centrifuged, and the precipitates were collected. After being washed with absolute ethanol and deionized water 3 times each, the precipitates of chitosan microspheres (CSMs) were freeze-dried and stored at room temperature for subsequent studies.

### 2.3. Preparation of Cyclodextrin-Grafted Chitosan Microspheres (CSM-g-CD)

Chitosan microspheres (CSMs) were surface-modified with β-cyclodextrin groups with a reaction between the amino groups of chitosan and the carboxyl groups of carboxymethyl-β-cyclodextrin (CMCD) mediated with hydrochloride (EDC) and N-hydroxysuccinimide (NHS). Briefly, 0.45 g of CMCD was dissolved in 10 mL of deionized water, and then 0.396 g EDC and 0.242 g NHS were added to the CMCD solution and reacted at 25 °C with stirring for 12 h. The activated CMCD solution was then added gradually to the CSM suspension containing 0.12 g CSM and reacted at 25 °C with stirring at 100 rpm for 24 h. The products of cyclodextrin-grafted chitosan microspheres (CSM-g-CD) were obtained after centrifugation and washing the precipitates with deionized water, followed by freeze-drying.

### 2.4. Preparation of Gelatin-Modified Responsive Microspheres (CSM-g-CD-Gel)

To obtain responsive microspheres, gelatin was attached to the surface of CSM-g-CD through dynamic host–guest interactions between β-cyclodextrin (β-CD) groups and aromatic residues in gelatin. Briefly, 0.4 g of gelatin was dissolved in 10 mL of deionized water under stirring at 100 rpm at 37 °C to obtain a 4% (*w*/*v*) gelatin solution, which was sterilized by filtration through a 0.45 µM filter. Then, after autoclaving, 0.5 g of CSM-g-CD microspheres was added to the gelatin solution and reacted under stirring at room temperature for 3 h. The resultant reaction mixture was centrifuged at 2000 rpm for 5 min, and the precipitates were retained. The unreacted gelatin molecules on the microsphere surface were removed by washing with deionized water. The modified responsive microspheres were labeled CSM-g-CD-Gel.

### 2.5. Physical Characterization of Chitosan Microspheres

#### 2.5.1. SEM

Chitosan microsphere samples in the wet state were frozen in a −20 °C freezer and then dried in a vacuum freeze dryer. Samples were surface gold sprayed for 100 s, and the morphologies of the appearance of the samples at an accelerating voltage of 20.0 kV were observed using an environmental scanning electron microscope (Quanta 200, FEI, Hillsboro, The Netherlands) and photographed for preservation.

#### 2.5.2. FTIR

The CSM, CSM-g-CD, CSM-g-CD-Gel, and AD-treated CSM-g-CD-Gel as well as gelatin were tested using the single reflection level smart ATR accessory of a Fourier transform micro infrared spectrometer (Vertex 70, Bruker, Germany). β-CD powder and AD powder were then tested using FTIR tableting, and the sample powder was thoroughly ground with an appropriate amount of KBr in a quartz mortar and poured into a tableting machine to squeeze into tablets. The transmittance of the samples at different wavenumbers was scanned in the range of 500~4000 cm^−1^, and infrared spectra were obtained and aligned based on data mapping.

#### 2.5.3. 2D NMR

A ^1^H NOESY NMR test was performed on CSM-g-CD-Gel at 37 °C to confirm the interaction between the aromatic residues of gelatin and β-CD groups in CSM-g-CD-Gel.

#### 2.5.4. Cytocompatibility Evaluation

For the nondirect contact cytotoxicity test, the CSM leaching solution was prepared according to ISO10993-5. After autoclaving, CSM and CSM-g-CD-Gel were soaked in cell culture medium at different concentrations (0.5, 1.0, and 2.0 mg/mL) for 72 h at 37 °C. The supernatant was collected as the extract after centrifugation at 10,000 rpm for 5 min. The extract was sterilized by filtration through a 0.22 μm filter before use. L-02 cells were seeded in 96-well plates at a density of 1 × 10^4^ cells/well and incubated overnight at 37 °C in a 5% CO_2_ atmosphere. Then, the aspirated and discarded medium was replaced by the extract solution, and the cell culture medium was used as a negative control. After 1, 2, and 3 days of culture, CCK-8 (Dojindo, Kumamoto, Japan) reagent was added to the plates at 10 µL/well and incubated at 37 °C for 30 min after gentle shaking for 5 min. The OD value of the solution in the well plate was measured at 450 nm using a multifunctional microplate reader (Multiskan, Waltham, MA, Thermo Fisher Scientific, USA). For direct contact cytotoxicity evaluation, cells were seeded in a mixture of microsphere suspensions at concentrations of 0.5, 1.0, and 2.0 mg/mL, and the cytotoxicity evaluation was performed according to the procedure described above. Cell viability was calculated by referring to the following formula, where *OD_S_*, *OD_B_*, and *OD_N_* are the *OD* values of the sample, blank control, and negative control, respectively.

(1) $$\mathrm{cell}\;\mathrm{viability}=\frac{OD_{S}-OD_{B}}{OD_{N}-OD_{B}}\times 100\%$$

#### 2.5.5. Cell Adhesion and Distribution on Microspheres

To evaluate the adhesion of cells to the above-prepared microspheres, CSM and CSM-g-CD-Gel were added to the cell culture medium to prepare a suspension of 2.0 mg/mL. The microsphere suspension (1 mL) was first mixed with 500 µL of cell culture medium containing 4 × 10^4^ L-02 cells for adequate contact. After that, the mixture was gently transferred to a 24-well plate and incubated at 37 °C in a 5% CO_2_ atmosphere. The adhesion of L-02 cells to the microsphere surface was examined under a light microscope after incubation for 4, 24, and 48 h. To further observe the growth and distribution of cells on the microsphere surface, cells were fluorescently stained. Nuclei of L-02 cells were stained using Hoechst 33,258 dye with blue fluorescence excitation using a mercury lamp as the excitation light source and a UV filter. The cell membrane of L-02 cells was stained using DIL dye, with a green filter for red fluorescence excitation. The cells were then incubated for 4, 24, and 48 h before observation under a fluorescence microscope.

#### 2.5.6. Responsive Detachment of Cells from CSM-g-CD-Gel Microspheres

L-02 cells were seeded onto responsive microspheres (CSM-g-CD-Gel) and cultured at 37 °C in a 5% CO_2_ atmosphere for 48 h. Adamantane (AD) solution at a concentration of 1.5% (*w*/*v*) was added to the culture and incubated for 5 min and 30 min, respectively. Then, the adhesion of cells on the microspheres was observed under a light microscope. To further verify the detachment of cells, cells were stained with Hoechst 33,258 dye and observed under a fluorescence microscope.

### 2.6. Statistical Analysis

Quantitative data are expressed as the arithmetic mean ± standard deviation (SD). All quantitative results were obtained from at least triplicate samples. The difference between groups was tested with *t* test. *p* < 0.05 was considered statistically significant, and *p* < 0.001 was considered highly statistically significant.

## 3. Results and Discussion

### 3.1. Morphological Analysis of the Microspheres

**Figure 1:**
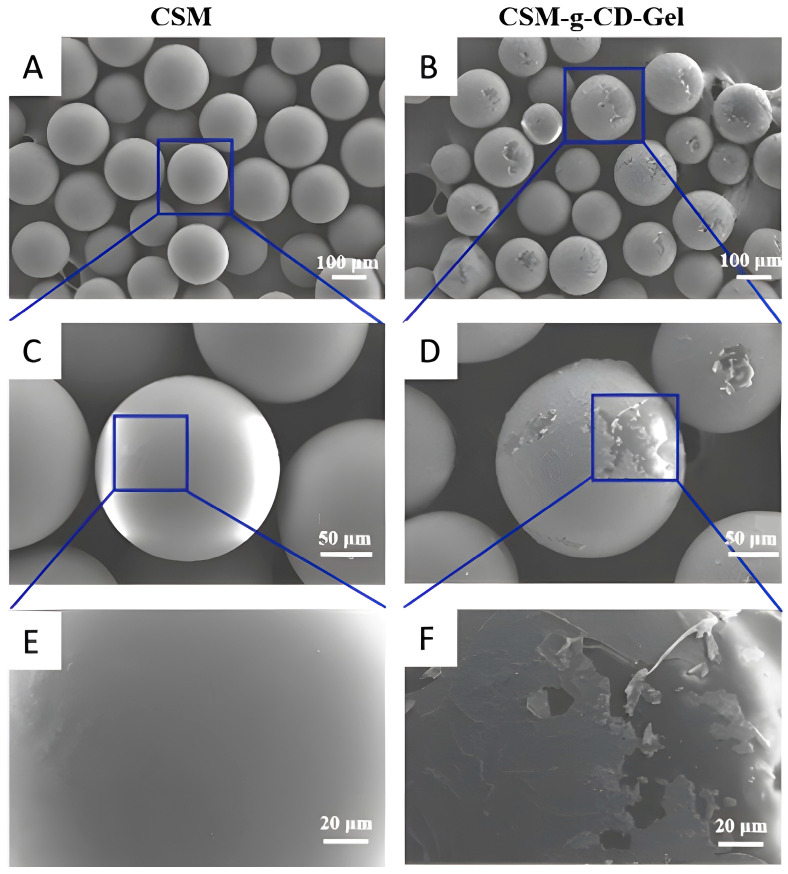
SEM images of CSMs (**A**,**C**,**E**) and responsive microspheres (CS-g-CD-Gel) (**B**,**D**,**F**); (**C**,**E**) and (**D**,**F**) are partially enlarged images of (**A**) and (**B**), respectively.

The surface morphologies of the microspheres were observed with SEM, and the results are shown in Figure 1. Figure 1A,C,E are the SEM images of CSM, and Figure 1B,D,F are the SEM images of CSM-g-CD-Gel. The surface of the CSM microspheres is smooth and flat, while the surface of the CSM-g-CD-Gel microspheres is relatively rough. It can be more clearly seen in the partially enlarged image (Figure 1F) that the surface of CSM-g-CD-Gel was wrapped with a thin layer of substance. It is speculated that this layer of substance might be gelatin, which attached to the microsphere surface by forming inclusion complexes with β-cyclodextrin groups through host–guest interactions.

### 3.2. FTIR Analysis of the Microspheres

**Figure 2:**
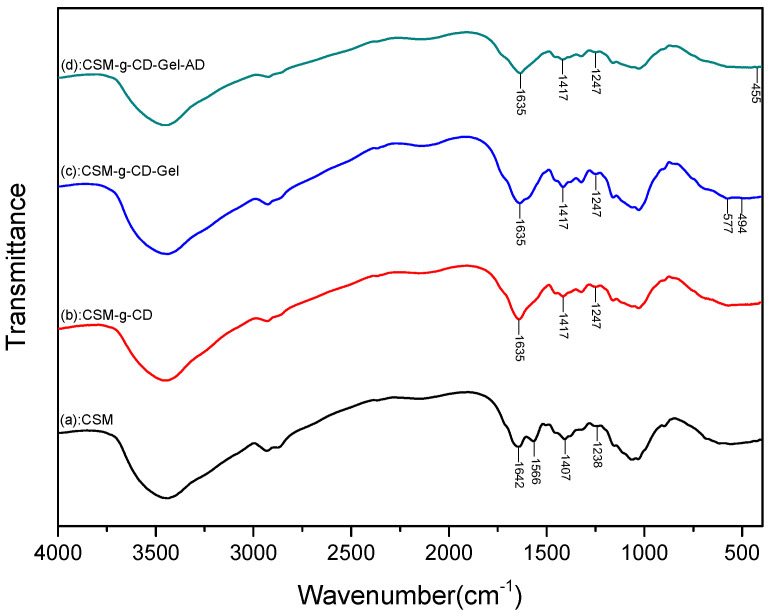
FTIR spectra of CSM (a), CSM-g-CD (b), CSM-g-CD-Gel (c), and CSM-g-CD-Gel-AD (d). CSM: chitosan microspheres; CSM-g-CD: CSM modified with β-CD groups; CSM-g-CD-Gel: CSM-g-CD modified with gelatin; CSM-g-CD-Gel-AD: CSM-g-CD-Gel after AD treatment.

To verify the grafting of β-cyclodextrin groups on the surface of chitosan microspheres as well as the subsequent modification of gelatin, the materials were characterized using FTIR, and the spectra are shown in Figure 2. In the spectrum of CSM, the large absorption peak at 3434 cm^−1^ is attributed to the stretching vibration of -OH and -NH groups on the molecular chain of chitosan. The peak at 2875 cm^−1^ is assigned to the -CH_2_ stretching vibration. The absorption peak at 1642 cm^−1^ is due to the C=O stretching vibration of the amide I bond, while the absorption peak at 1566 cm^−1^ is due to the N-H stretching vibration of the amide II bond [9]. The peak at 1382 cm^−1^ is assigned to the N-H absorption of the amide III bond. The peak at 1157 cm^−1^ is the stretching vibration of the glucoside C-O-C bond between the chitosan monomers and glucose. The peaks at 1080 cm^−1^ and 1028 cm^−1^ are the characteristic stretching peaks of polysaccharide C-OH. In comparison to the spectrum of CSM, the amide I peak of CSM-g-CD at 1642 cm^−1^ was shifted to 1635 cm^−1,^ and the peak at 1566 cm^−1^ disappeared, which resulted from the formation of a new amide bond between the amino groups on chitosan and the carboxyl groups on CMCD. The formation of new absorption peaks at 1417 cm^−1^ and 1247 cm^−1^, derived from CMCD [10], was also observed in the spectrum of CSM-g-CD, indicating that CMCD was successfully grafted onto the chitosan microspheres to form CSM-g-CD. It was further found that in the spectrum of CSM-g-CD-Gel, new peaks at 577 cm^−1^ and 494 cm^−1^ were present, which were obtained from gelatin [17]. Combined with the SEM results, it can be concluded that gelatin successfully reacted onto the microspheres to form CSM-g-CD-Gel. In addition, the CSM-g-CD-Gel microspheres were also subjected to IR analysis after adamantane (AD) treatment. As shown in Figure 2, compared with CSM-g-CD-Gel, the peak intensities at 577 cm^−1^ and 494 cm^−1^ of gelatin were obviously weakened, and a new peak appeared at 455 cm^−1^, which might be the skeleton C-C-C bond deformation vibration of AD according to the literature reports [18]. This result suggests that gelatin molecules on the surface of the CSM-g-CD-Gel microspheres were replaced by AD molecules because of the higher affinity between the AD and CD groups than between gelatin and CD [19].

### 3.3. 2D NMR Analysis

**Figure 3:**
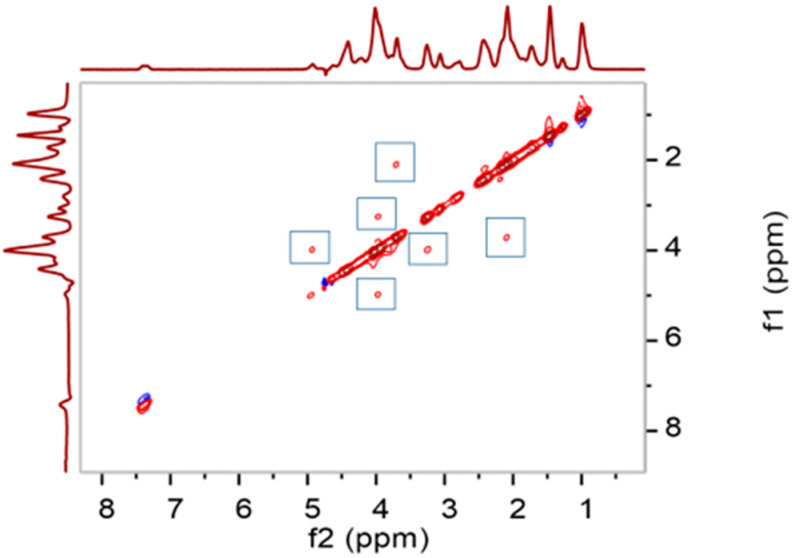
The 2D ^1^H NOESY spectrum of CSM-g-CD-Gel.

Two-dimensional (2D) ^1^H NOESY NMR was used to verify the binding of gelatin to the microspheres via the formation of inclusion complexes between aromatic residues in gelatin and β-CD groups on the surface of the microspheres. As shown in Figure 3, multiple symmetrical cross-signal regions can be seen in the spectrum of CSM-g-CD-Gel, indicating that the aromatic hydrogens of gelatin and the alkane hydrogens of β-CD are spatially close to each other, and their atomic nuclei produce a NOE effect, thereby demonstrating that the aromatic residues of gelatin formed inclusion complexes with the β-CD groups through host–guest interactions; thus, gelatin molecules were successfully linked to the surface of the microspheres [20].

### 3.4. Cytocompatibility

**Figure 4:**
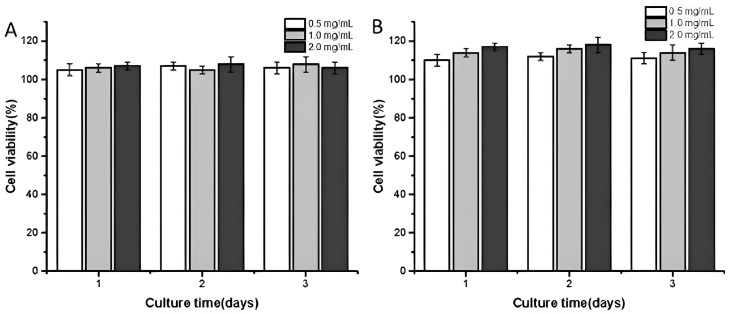
CCK-8 cytotoxicity analysis of chitosan microspheres and smart microspheres (**A**) CSM; (**B**) CSM-g-CD-Gel.

To analyze the cytocompatibility of chitosan microspheres and responsive microspheres, CCK-8 was employed for cytotoxicity experiments. L-02 cells were seeded on CSMs and CSM-g-CD-Gel at concentrations of 0.5 mg/mL, 1.0 mg/mL, and 2.0 mg/mL. As shown in Figure 4, in general, the cell viability of all the samples with different concentrations at different time intervals was more than 100%, which indicated that both the CSM and CSM-g-CD-Gel microspheres did not cause toxic effects on the cells and had good cytocompatibility. Notably, it was found that the cell viability on the responsive microspheres was slightly higher than that on the CSMs. Meanwhile, it was also found that the cell viability on CSM-g-CD-Gel had a tendency to increase with the material concentration. This may be related to the fact that gelatin on responsive microspheres can promote cell adhesion and growth, which has been well-recognized previously [21,22,23].

### 3.5. Cell Adhesion and Distribution on CSM-g-CD-Gel

**Figure 5:**
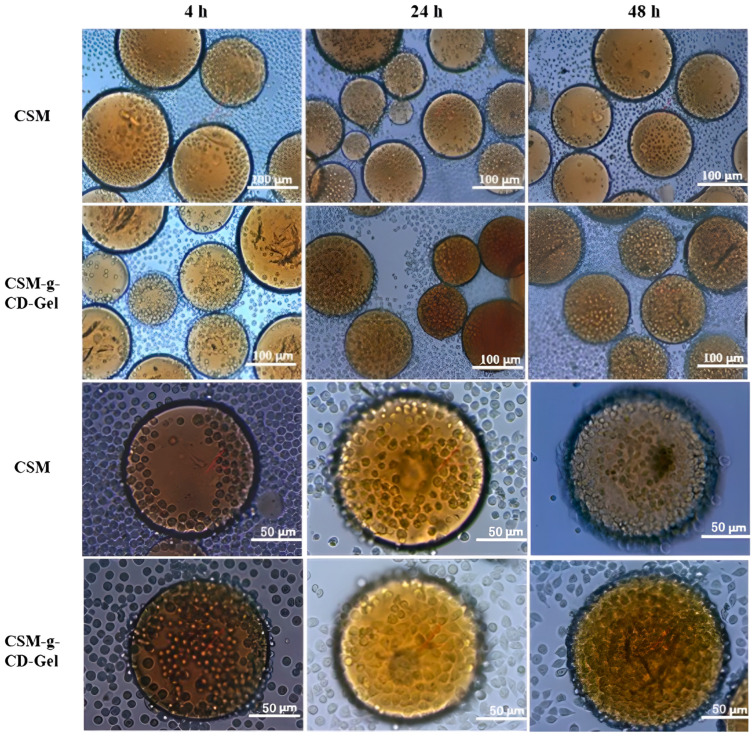
Optical microscopy images of the attachment and growth of L-02 cells cultured on CSM and CSM-g-CD-Gel for 4 h, 24 h, and 48 h. The scale bar for the upper two rows of images is 100 µm. The scale bar for the lower two rows of images is 50 µm.

The adhesion and distribution of L-02 cells on CSM and CSM-g-CD-Gel are shown in Figure 5. It was observed under a light microscope that, with increasing incubation time, the number of cells adhering to the surface of the microspheres gradually increased. In the first 4 h, only a small number of cells adhered to the microsphere surface, and the number of cells began to increase at 24 h. When the incubation time increased to 48 h, most of the microsphere surface was covered with cells. The number of cells on the surface of the CSM-g-CD-Gel was greater than that on the surface of the CSM, especially at 4 h; that is, at the cell attachment stage, a large number of cells adhered to the surface of the responsive microspheres. This is probably because there are gelatin molecules on the surface of the responsive microspheres that promote cell adhesion. This is also consistent with the results of the cytotoxicity tests.

**Figure 6:**
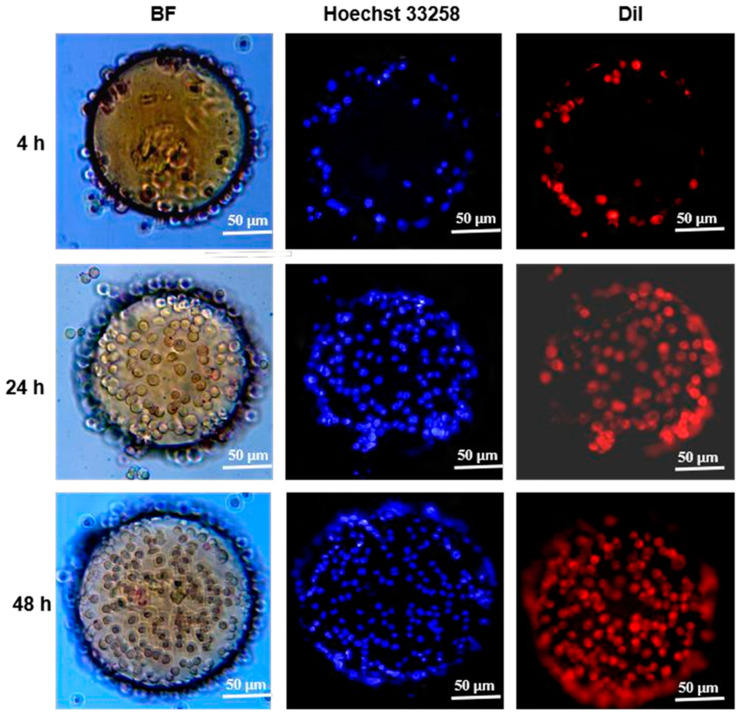
Fluorescence microscopy image of cell morphology and distribution on CSM-g-CD-Gel microspheres for 4 h, 24 h, and 48 h. Hoechst 33,258 stained nucleus; Dil stained cell membrane. The scale bar is 50 µm.

To further analyze the cell distribution as well as the cell morphology on CSM-g-CD-Gel, the cells on the microspheres were fluorescently stained for nuclei and cell membranes, and the results observed under a fluorescence microscope are shown in Figure 6. In general, the results were consistent with those observed under an ordinary light microscope, and the number of cells adhering to the microspheres increased as the incubation time increased. At the beginning of the culture (4 h), the cells were distributed only at the edges of the microspheres, and subsequently, the cells began spreading, migrating, and growing over the microspheres. At 48 h of culture, the cells had spread over the microspheres. Meanwhile, it could also be seen that the cells had an intact structure and morphology from the blue fluorescence-labeled nucleus and the red fluorescence-labeled cell membrane. These results further indicate that the CSM-g-CD-Gel microspheres are favorable for cell adhesion and proliferation and can maintain the morphology of cells. It is well-known that collagens are crucial structural components of the extracellular matrix that provide cells with abundant adhesive sites and affect cell fate. Peptide sequences such as RGD in collagens can act as specific ligands to integrins, a family of transmembrane proteins of cells that facilitate cell adhesion, growth, proliferation, and communication [24,25,26]. Gelatin, as a product of partial hydrolysis of collagens, possesses remarkable biological effects similar to collagens and thus has been widely applied in the biomedical field. The specific peptide sequences retained by gelatin can also act as anchoring sites for cells and promote cell adhesion, growth, proliferation, and communication [27,28]. Therefore, through the interaction between gelatin and the cells, CSM-g-CD-Gel microcarriers displayed superior abilities to support cell adhesion and expansion in vitro.

### 3.6. Responsive Detachment of Cells

**Figure 7:**
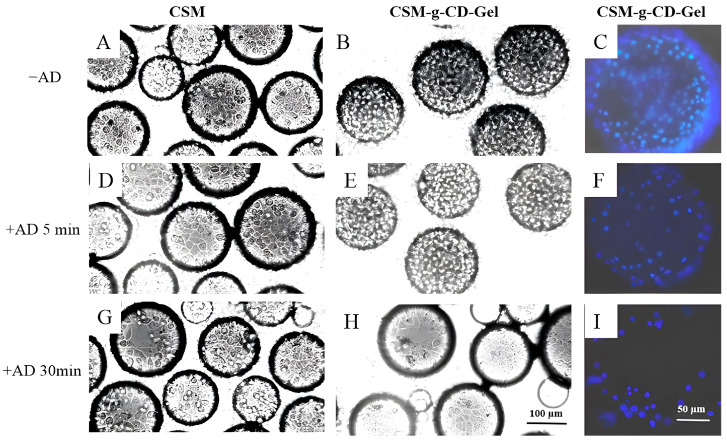
Cell detachment from CSM-g-CD-Gel after treatment with amantadine (**A**,**D**). The scale bar for (**A**,**B**,**D**,**E**,**G**,**H**) is 100 µm. The scale bar for (**C**,**F**,**I**) is 50 µm.

Cell detachment from the CSM-g-CD-Gel microspheres was achieved by adding AD molecules to the culture. The results are shown in Figure 7. When the cells were cultured on the microspheres for 48 h, the cells spread well on both CSM and CSM-g-CD-Gel (Figure 7A,B). When AD molecules were added and reacted for 5 min and 30 min, the number of cells on CSM was not significantly different from that before AD treatment, as shown in Figure 7D,G. In comparison, the number of cells on CSM-g-CD-Gel decreased after 5 min of AD treatment, as shown in Figure 6E. Furthermore, after 30 min of AD treatment, the number of cells on the CSM-g-CD-Gel microspheres was significantly decreased (Figure 7H), indicating that a large number of cells had detached from the microspheres. To further verify the cell detachment, the nuclei of the cells on microspheres were stained to show the decrease in cell number on microspheres more clearly after AD treatment, as shown in Figure 7C,F,I. The AD-responsive cell detachment from the CSM-g-CD-Gel microspheres is due to the competitive binding of AD molecules to the CD cavities, leading to the release of gelatin molecules, which tightly bind cells. The cells detached together with the gelatin released from the microspheres. The above results indicate that the CSM-g-CD-Gel microspheres can be used as smart microcarriers for cell culture and enzyme-free cell detachment.

## 4. Conclusions

The construction of responsive microcarriers based on chitosan microspheres and their application in cell culture and enzyme-free detachment were studied in this work. First, chitosan microspheres were prepared with the emulsification crosslinking method, and through optimization of the preparation conditions, solid chitosan microspheres (CSMs) with spherical shapes and particle sizes ranging from 100 to 300 µm were obtained. This was followed by the successful grafting of β-cyclodextrin (β-CD) groups on the surface of CSM via an EDC/NHS-mediated acylation reaction and further gelatin modification to obtain CSM-g-CD-Gel utilizing the host–guest interactions between β-CD cavities and the aromatic residues of gelatin. SEM, FTIR, and 2D 1H NMR were employed to characterize the microspheres. The CSM-g-CD-Gel microspheres exhibited good cytocompatibility, allowing cells to attach and grow over the microsphere surface and maintain cell morphology. Finally, nonenzymatic detachment of cells together with gelatin from the CSM-g-CD-Gel microspheres can be achieved by the addition of AD based on the competitive binding of AD molecules to the CD cavities. Overall, CSM-g-CD-Gel microspheres may potentially be applied as smart microcarriers for 3D cell culture and enzyme-free detachment.

## Data Availability

Data will be made available upon request.
